# Investigating the modulation of stimulus types on language switching costs: Do semantic and repetition priming effect matter?

**DOI:** 10.3389/fpsyg.2023.1090744

**Published:** 2023-04-17

**Authors:** Qinfang Shen, Yixin Chen

**Affiliations:** ^1^Department of Theoretical and Applied Linguistics, University of Cambridge, Cambridge, United Kingdom; ^2^Institute of Education, University of College London, London, United Kingdom

**Keywords:** language switching costs, bilingual language production mechanism, L2 inhibitory control, asymmetrical switching costs, priming effect, lexical access, language style

## Abstract

**Introduction:**

In the present study, I investigated the influence of stimulus types on bilingual control in the language switching process. The commonly employed stimuli in language switching studies – Arabic digits and objects – were compared to further investigate the way in which inhibitory control could be modulated by semantic and repetition priming effects. The digit stimuli have two unique characteristics in the language switching paradigm, for example, they are present repeatedly and are semantically related to each other, compared with pictural stimuli. Thus, these unique characteristics might influence the operation of inhibitory control in bilingual language production, modulating the size and asymmetry of switching costs.

**Methods:**

Two picture control sets were set up to match those characteristics: (1) a semantic control set, in which picture stimuli belong to the same category group, such as, animals, occupations or transportation and specific semantic categories were presented in a blocked condition; and (2) a repeated control set, in which nine different picture stimuli were repeatedly presented like the Arabic digits from 1 to 9.

**Results:**

When comparing the digit condition and the standard picture condition, analyses of naming latencies and accuracy rates revealed that switching costs were reliably smaller for digit naming than for picture naming and the L1 elicited more switching costs for picture naming than for digit naming. On the other hand, when comparing the digit condition and the two picture control sets, it was found that the magnitude of switching costs became identical and the asymmetry in switching costs became much smaller between the two languages.

## 1. Introduction

One of the most amazing abilities of fluent bilinguals is to seamlessly switch between two languages without breaking a sweat. According to previous literature of bilingual language production and comprehension, semantic representations simultaneously activate two lexicons of bilinguals even when speaking in one of the languages (Poulisse and Bongaerts, 1994; Green, 1998; Costa et al., 1999; Dijkstra and van Heuven, 2002). This raises a question here as to why lexicon co-activation does not lead to substantial non-target language intrusion when speaking in the target language, for example, previous evidence suggests that bilinguals make few language errors. This finding led Green (1998) to suggest that there must be a language control mechanism to regulate concurrent language coactivation in a way that it suppresses non-target language activation to guarantee the speech production in the target language.

One important issue here is as to why the language co-activation does not result in massive intrusions from the non-target language when speaking in the target language, for instance, previous evidence has shown that bilinguals rarely make language errors (Poulisse and Bongaerts, 1994). These findings have led Green (1998) to argue that a language control mechanism must be in place to mediate the concurrent language co-activation, which inhibits the activation of the non-target language in order to produce speech in the target language. So far, Green’s Inhibitory Control model (the IC model, hereafter) has received compelling evidence from the language switching paradigm in which participants are asked to name objects or Arabic numbers in either their first or second language (e.g., Meuter and Allport, 1999). This naming condition forms two types of trials: (1) the stay trial in which the naming language in the current trial is the same as the preceding trials; and (2) the switch trial in which the current response language differs from the one used in the previous trial. The typical finding is that switch trials resulted in slower naming latencies and more naming errors than stay trials did. The naming latency difference between switch and stay trials has been referred to as so-called “language switching costs.” However, previous language switching studies have not reached consistent results regarding the size and (a)symmetry of switch costs due to various methodological differences such as different stimulus types (pictures and digits) and a variety of preparation time. Focusing on stimulus types, the present study aimed to examine whether and how this methodological difference modulate the size of and (a)symmetry in switching costs, using a cued language switching paradigm.

It is both empirically and theoretically important to investigate the impact of stimulus types on language switching. First, it could shed light on whether or not language switching studies using various stimulus materials are comparable. This is of great importance considering that the size and (a)symmetry of switching costs differ greatly among studies using different types of stimuli, that is, numerical digits and pictures (or objects; e.g., Costa and Santesteban, 2004; Costa et al., 2006; Philipp et al., 2007; Verhoef et al., 2010; Guo et al., 2011; Mosca and Clahsen, 2015; Chang et al., 2016). Furthermore, some studies even challenged the presence of an inhibitory control mechanism and its proposed persisting inhibition effect in bilingual language production as they did not observe asymmetrical switching costs as empirical evidence for inhibition. However, the present study intended to seek evidence of the modulation of stimulus types on language switching costs, and therefore the absence of asymmetrical switching costs does not necessarily deny the mechanism of inhibitory control. Second, it has been well established that many methodological differences that go beyond stimulus differences, such as speakers’ language proficiency and the length of preparation time, account for a lack of asymmetrical switching costs, leaving stimulus differences untouched. Therefore, a thorough understanding of how stimulus differences affect language switching process allows for unambiguity and precision in the future research design, as well as a clear indication of whether and how the language production processing stage could play a role in bilingual language control.

## 2. Literature review

### 2.1. General findings on language-switching paradigm

In the trial-by-trial language-switching task, participants are required to name items (e.g., standardized black-and-white line drawings or Arabic digits from 1 to 9) in either their first or second language. The language in which stimuli are expected to be named depends on a colour cue (usually the colour of the background screen), varying from trial to trial. This gives rise to different types of trials. For example, in the non-switch (or stay) trial, participants name the stimulus in the same language as the one used in the preceding trial. In contrast, in the switch trial, participants name the stimulus in a different language from the one used in the preceding trial. The general finding in this context is that participants’ naming performance is impaired in the switch trial than in the stay trial. Specifically, switch trials result in slower naming latencies and more naming errors. The calculation of subtracting naming latencies of switch trials from non-switch trials is called “language switching cost.” These switch costs have also been found in the switching paradigm that does not involve linguistic processes such as the task-switching paradigm (e.g., Meiran, 1996; Rogers and Monsell, 1996; Monsell, 2003; Kiesel et al., 2010).

The first influential study to examine the consequences of the cross-language competition and the possibility of bilingual language control was by Meuter and Allport (1999). In their study, proficient (but not balanced) bilinguals performed in the numeral switching task, with much theoretical underpinning borrowed from task-switching theories. As said, they were required to name the Arabic numerals in either their first or second language according to colour cues. The authors hypothesized that based on the task set inertia hypothesis (Allport et al., 1994), one could predict that the dominant task should result in larger switch costs than the nondominant task. This is because the dominant task needs to be more suppressed in order to perform the nondominant task. As a result, when subsequently switching into the dominant task, more time and effort are needed to re-activate the dominant task. In contrast, switch costs should be smaller when switching to the less dominant task, due to the less suppression exerted on the weaker task in the preceding trial. This was exactly what the authors observed.

The results showed that naming latencies of switch trials were slower than those of stay trials and L2 switch trials resulted in faster naming latencies than L1 switch trials did, pointing to the asymmetry in switching costs. This suggests that switching from the weaker language (e.g., L2) to the more dominant language (e.g., L1) was more costly than the other way around, resulting in an asymmetrical switching cost. The finding of an asymmetrical switching cost is perfectly interpreted as evidence of the IC model. As mentioned above, the IC model assumes that the amount of inhibitory control exerted on a language is proportional to its strength; in other words, the more dominant or stronger the language, the greater the inhibition is exerted. Following this line of logic, the stronger L1 should be more suppressed when it serves as the non-target language in the L2 switch trial. As a consequence, it should take more time to overcome this inhibition when switching into the L1, that is, language re-activation becomes more difficult because of the stronger inhibition, resulting in the observed asymmetrical switch costs.

Meuter and Allport (1999) also proposed that relative proficiency levels of bilinguals’ two languages should affect the degree of switching cost asymmetry. To test this assumption, the researchers divided their participants into two groups according to their L2 proficiency levels; one group showed more L1 dominance while the other was relatively balanced bilinguals. It was found that the unbalanced participants continued to show the asymmetrical switch costs, while the balanced group did not, which suggests that the language proficiency could modulate the asymmetry in switching costs and further confirms the assumption that inhibition applied to an unintended language is proportional to its relative strength (Costa and Santesteban, 2004; Gollan and Ferreira, 2009; Calabria et al., 2012).

Nevertheless, there has been inconsistency in the research findings on the pattern of switching costs (including both size and (a)symmetry), thus leading authors to challenge the involvement of inhibitory control mechanisms in bilingual language production. However, it appears premature to assume that the absence of asymmetry reflects a lack of inhibitory control, as both participant- and task-related variables might influence the results. For instance, to explore how a participant-related factor (i.e., language proficiency level) affects the IC mechanism, Costa et al. (2006) observed the language switching performance of trilingual when switching between their L1 and L2 and L2 and L3 (note that these participants were highly proficient at their L2 but less proficient at their L3). Surprisingly, symmetrical switching costs were reported in their two experiments regardless of the dominance of the two languages involved in switching, replicating their previous study (Costa and Santesteban, 2004), which led them to argue that differences in proficiency levels do not necessarily result in asymmetry in switching costs for highly proficient or balanced bilinguals. This is because, as they suggested, when performing language switching tasks, highly proficient bilinguals do not rely on inhibition of the unintended language because they develop a so-called “language-specific selection” mechanism where bilingual language control is not required (Costa et al., 1999).

These observations may lead one to ask, “is inhibition only called upon when unbalanced bilinguals switch between two languages?” or “can we therefore conclude that the presence of inhibition depends on the proficiency level of bilingualism?” (Bobb et al., 2013, p. 494). The answer to these questions might be no, as the proficiency difference is not the only consideration affecting switching costs, in other words, task-related variables, i.e., preparation effect, stimulus differences, language similarity, and so on, could also be an alternative explanation of the presence of symmetrical switching costs in Costa et al. studies. The rationale for such as a claim is that the experiment settings in Costa et al.’s (2006) study differed greatly from Meuter and Allport’s (1999), allowing for a longer preparation effect between the colour cue and the presence of pictorial stimulus (the cue-to-stimulus interval, CSI). This preparation effect, as measured by various CSIs, has received scholarly attention in both language and task switching research and is thought to modulate the size and (a)symmetry of switching costs (e.g., Meiran, 1996; Costa and Santesteban, 2004; Philipp et al., 2007; Verhoef et al., 2010; Declerck et al., 2012).

Despite the fact that no convergent evidence has been shown about how CSIs exactly affect the size and (a)symmetry of switching costs, largely due to methodological differences and a great variation in research design, investigating the interaction between preparation effect and switching costs is fruitful as it deepens our understanding of how inhibition control functions in different stages of the language switching process and what kinds of factors could affect it. However, little is known about how other task-related variables, such as stimulus types, modulate inhibition during language control. Some studies used digits (e.g., Meuter and Allport, 1999; Jackson et al., 2001), whereas others used pictures (e.g., Mosca and Clahsen, 2015) or a limited number of pictures (e.g., Costa and Santesteban, 2004; Costa et al., 2006). According to Levelt et al.’s (1999) word production model, repeated access to semantic representations at the conceptual preparation stage can facilitate the activation of their lemmas. One might ask, in digit naming, whether and how enhanced activation at the concept and lemma selection stages could modulate the functionality of the IC model.

It is worth noting that in both Costa and Santesteban (2004) and Costa et al. (2006), only ten pictures were used for hundreds of trials, and this practice effect may have resulted in a decrease and the symmetry of switching costs, as what they observed. It can therefore be argued that proficiency or language dominance alone cannot interpret inconsistencies in switching costs. Therefore, a more detailed investigation of other factors should be conducted. Furthermore, a systematic investigation of stimulus type could reveal a complex picture of the inhibitory control process in terms of its processing stages and mechanism, as well as account for observed non-convergent patterns of asymmetries and strengths in switching costs.

### 2.2. The effect of stimulus types on switching cost: Digit vs. picture

Previous language switching studies mainly used two types of stimuli: Arabic digits from 1 to 9 (e.g., Meuter and Allport, 1999; Jackson et al., 2001; Philipp et al., 2007; Guo et al., 2011) and random objects (pictures) (e.g., Costa and Santesteban, 2004; Costa et al., 2006; Verhoef et al., 2010; Mosca and Clahsen, 2015; Chang et al., 2016). Therefore, it might be problematic to compare directly language switching studies because of the methodological differences. It should be noted that picture and digit stimuli differ on various levels. First, according to Herrera and Macizo (2010), the digits represent a specific semantic group, which is not the case for the picture stimulus. It has been well documented that in the blocked semantic naming paradigm, longer naming latencies have been observed when pictures were semantically related to each other than when they were unrelated (e.g., Oppenheim et al., 2010). This impaired performance in the semantic blocking context has been referred to as the “semantic interference effect” or “semantic blocking effect” (e.g., Cipolotti et al., 1995; Herrera and Macizo, 2010).

One could argue that if picture stimuli belong to the same semantic categories, then the naming latencies in the stay and switch trials should be slower since the coactivation of semantically-related items in two languages competes for selection. However, this may not be the case in the language switching paradigm because language membership changes in switching trials might lead to the disappearance of the semantic interference effect (e.g., Green, 1998; Lee and Williams, 2001; Runnqvist et al., 2012), which will be further discussed in the General Discussion section. However, in stay trials, according to Howard et al. (2006), when a sequence of pictures forms a single semantic group, two effects play a role in parallel. First, participants might be able to anticipate the semantic category of the picture stimulus in advance; second, a short-term facilitatory semantic priming effect (at the conceptual level) from the previous trial should facilitate the selection of the target word. This short-term semantic facilitation effect has been observed in previous studies, for instance, Wheeldon and Monsell (1994) found that producing a word ‘dog’ transiently speeded up the subsequent naming of a picture of a ‘cat’. This argument is also in line with findings that bilingual language control occurs at different lexical processing stages such as, phonological, conceptual and orthographical selection stages (e.g., Declerck et al., 2015; Zhang et al., 2020).

Another evidence for this short-lasting semantic facilitation effect comes from Damian and Als (2005). In their study, participants were required to name pictures within homogeneous and heterogeneous contexts four times (presentation cycle 1–4). The results showed that the semantic interference effect was absent on the first presentation of each item, which emerged thereafter, remaining stable for the remainder of the presentations, and there is a semantic facilitative effect characterized by faster object naming latencies in the first presentation cycle of the homogeneous block. Navarrete et al. (2014) took this issue even further and they explored the effect of within-category semantic distance (within-category semantically close vs. far) on the pattern of facilitation and interference effects. The same pattern of result was replicated: semantic distance did not modulate the facilitation effect in the first presentation cycle.

In their subsequent study (Navarrete et al., 2014, p. 259), they redesigned the traditional blocked picture naming paradigm in which the semantically related pictures were present repeatedly per block and a consequential design was introduced: (1) “each picture was presented once per block” and (2) “blocks were repeated multiple times.” Strikingly, their results showed that (1) the semantically related condition facilitated picture naming and (2) the semantic interference effect was only observed when pictures were presented multiple times per block. They argued that this facilitation effect might result from a trade-off between lexical interference and semantic facilitation. That is, the magnitude of the conceptual facilitation might override that of the lexical interference. For instance, participants might notice the shared semantic features of the items in homogeneous contexts and use this knowledge to predict the category of other items in this cohort. Belke et al.’s (2017) study further confirmed that this kind of facilitation effect was strategic in nature, and the blocked-cyclic paradigm allowed participants to bias the levels of activation of the semantic representations, resulting in a processing advantage for the members of a specific semantic category. This bias, according to Belke et al. (2017), operates at the conceptual level but not the lexical level.

Furthermore, another theoretical model that can possibly provide empirical evidence for the argument that semantic priming effect would affect switching costs comes from Kroll and Stewart’s (1994) Word-Concept Association model in bilingual language production. This model suggests that the strength of link between semantic concepts and lexical representations (or lemmas) differs from L1 to L2, that is, concepts have a stronger link to their corresponding lexical representations in L1 than L2. Following this logic, it could very well be that in switch trials, recovery from the inhibition of L1 is less time-consuming and effortless due to its enhanced concept activation that allows L1 lexical representations receive more activation from the concepts than L2 lexical representations. Then, it can be predicted that the (a)symmetry of switching costs can be affected when stimuli are semantically related. In sum, the results observed from these traditional semantic blocking studies might be taken as evidence that the semantic priming effect or semantic blocking effect formed in the digit naming in the language switching paradigm is likely to modulate the switching costs.

There is another difference between the digit and picture naming in the language switching paradigm. Specifically, language switching studies using digits as stimuli typically employ nine numerals repeatedly throughout the experiment, while studies using pictures employ unique objects in each trial or a certain number of pictures that are repeatedly presented fewer times. In this case, if the same stimulus such as a word or an object is presented several times within finite intervals, then it will be processed more efficiently at the subsequent occurrence (e.g., Hernandez and Reyes, 2002). Such facilitation has been referred to as the repetition priming effect. Following this logic, it is clear that the limited number of digit numerals (from one to nine) can cause a robust repetition priming effect relative to the picture stimulus that varies from trial to trial, thus modulating switching costs.

The third difference between picture and digit naming is that digits might be cognate (referring to a phonological overlap between languages) in two languages from the same language family such as, romance language. Studies have provided support for the claim that the cognate can facilitate bilingual picture naming (e.g., Costa et al., 2000; Hoshino and Kroll, 2008). This facilitation effect has been taken as evidence of phonological co-activation of two languages. In this case, in a bilingual group whose two languages belong to the same language family (e.g., English-German or English-Spanish bilingual speakers), numeric digits could have large phonological overlap between languages, which provides the possibility that cognate status may also make a difference to the switch costs. This is the case in both Verhoef et al. (2009) and Declerck et al. (2012), who reported that cognates in digital stimuli led to symmetrical switching costs in German–English and Dutch–English bilinguals’ performances. For instance, Verhoef et al. (2009) reported smaller switching costs with cognates, which is also the case in Declerck et al. (2012), who even reported symmetrical switching costs with cognates. It is also interesting to explore whether the digital effect could extend to language pairs without cognate status, as in the present study (Chinese–English).

As shown in the review of the current body of research, there is not a consistent picture of switching costs in the language switching paradigm due to methodological differences especially in the stimulus variation across studies. In addition, so far, no systematic investigation has been conducted to examine the way in which these methodological differences modulate the size and (a)symmetry of switching costs. To such an end, the current experiment was designed to investigate this issue.

## 3. Research methodologies

This experiment explores whether and how stimulus types affect the language switching performance of proficient (unbalanced) Chinese–English bilinguals. To do so, participants’ naming latencies and accuracy rates in digit naming were compared to those in random picture naming, semantically related picture naming and repeatedly presented picture naming. This manipulation allows to explore the factors that may contribute to observation of digit effects in the language switching paradigm.

*Hypothesis 1*: Switching costs can be modulated by different types of stimuli, that is, switching costs might be different between the digit naming and picture naming.

*Hypothesis 2*: The semantic relationship between the stimulus can lead to a reduction and symmetry in switching costs.

*Hypothesis 3*: The repetition of the stimulus can reduce switching costs and lead to a symmetry in switching costs.

As argued in the literature review section, there are methodological differences between picture naming and digit naming in language switching studies. Specifically, studies employing pictures use unique stimuli for each trial, while studies employing digits use repeated numerals (from 1 to 9) during the experiment. Given different patterns of switching costs (i.e., symmetry and asymmetry in switching costs, presence or absence of switching costs) reported in these studies, the current experiment aimed to examine whether stimulus type differences are responsible for switching cost variance.

According to Declerck et al. (2012) and Liu and Chaouch-Orozco (2022), digit naming is different from (random) picture naming in the language switching paradigm in three aspects: (1) digits could formulate cognates when two languages share an alphabet system (i.e., English, Dutch, and German), which is not the case for pictural stimuli although some pictures might also have cognates between two languages (2) numerical digits are repeatedly presented and named for hundreds of times, and (3) digits are semantically related, formulating a digital concept group that makes co-activation of semantically-related concepts possible. Previous literature has shown that inhibitory control occurs at different processing stages, such as at the concept level (Bobb et al., 2013), at the lemma level (e.g., Green, 1998), at the phonology or orthography level (e.g., Declerck et al., 2012). Therefore, it is interesting to assume that these specific digital features modulate language control, thereby causing the inconsistency in the size and (a)symmetry of switching costs that reported in different studies using digits and pictures as their stimuli. Since the language combination in this study is Chinese and English which do not have any phonological overlap and cognates, only the last two features were examined.

By comparing standard picture naming to semantically related picture naming, one can test whether the semantic activation impact inhibitory control and language switching. Following the same logic, by comparing standard picture naming to repeated picture naming, one can test whether repetition priming impact inhibitory control and language switching. Once we got the results and confirmed hypotheses that these two effects indeed modulated language switching, it is rationale to argue that the stimulus type is a factor affecting language switching and one can call for an attention to future researchers that Arabic digits might not be ideal stimuli considering their mixed effects.

The comparison between the digit numbers (from 1 to 9) and the standard pictures in which picture stimuli are not unrelated and unrepeated allowed me to address the question of whether switching costs could be affected by stimulus types. Furthermore, as argued before, digits have two unique characteristics that (1) they are semantically related to each other and (2) they are repeatedly named during the experiment. To match these characteristics, two other picture sets were added: (1) the semantic control set in which picture stimuli are semantically related to each other, and (2) the repeated control set in which nine semantically unrelated pictures were repeatedly presented in this blocked condition like the numerals from 1 to 9. These two control sets aimed to investigate which digit characteristics could result in switching cost differences observed in the first comparison.

### 3.1. Participants

Twenty participants who were postgraduate students at the University of Cambridge participated in this experiment (13 males and seven females, mean age = 25.3). Participants were all right-handed and had normal or corrected to normal vision. They reported Chinese as their stronger first language (L1) and English as their weaker second language (L2). Despite that they started learning English at different ages (ranging from 3 to 12 years old; mean age = 8.1), all 20 participants received formal English training from their junior high school. In addition, all participants had taken the International English Language Test Systems (IELTS) for admission to the University of Cambridge and achieved over 7.5 overall scores. More importantly, most participants had been studying abroad (United Kingdom, United States, and Australia) for their undergraduate degree for more than 3 years, which means that they had much more opportunities to switch between English and Chinese. Finally, participants were paid (4 pounds) as compensation. Power analysis should be conducted in future work on language switching.

### 3.2. Materials and designs

As aforementioned, the aim of this experiment is to investigate the effects of digit characteristics on language switching costs and this exploration could shed light on the difference in switch costs observed in previous studies using different stimulus types (digits and pictures). The four stimulus sets were presented in different blocks, which consisted of (1) a pure digit block (Arabic digits from 1 to 9); (2) a standard picture block in which the object were unrelated and not presented repeatedly; (3) a control block with semantically-related pictures (e.g., animal: dogs/cats; career: firemen and soldiers); and (4) a control block with nine repeated pictures that were semantically unrelated.^1^ All the numeric stimuli were presented pseudo-randomly so that the same digit was not presented consecutively. All pictures were black-and-white line drawings.

The whole digit condition consisted of 61 Arabic digit stimuli. The first trial was a null switch trial, and therefore there were 60 trials in total. Half trials were to be named in Chinese and the other half in English. Crucially, there were two types of trials: (1) switch trials, in which the current stimulus was to be named in a different language from the preceding one (e.g., Chinese, English or English, Chinese); and (2) non-switch or stay trials, in which the current stimulus was to be named in the same languages as the previous one (e.g., Chinese, Chinese or English, English). The number of these two types of trials was balanced.

The standard picture block was composed by unrelated common objects that were selected from the International Picture Naming Project database. The semantically-related picture block comprised items related to animals, careers and transportations, respectively. Similar to the digit block, there were 61 pictures and 60 trials with equal number of language switches and repetitions in these two pictural blocks. Finally, other nine pictures formed the repeated picture set and were presented repeatedly within 60 trials. Each picture (2 cm high*1 cm wide) was presented at the center of the laptop screen. The block order was counterbalanced across participants, but the trial sequence in each block was kept fixed. The response language in each trial was indicated by a colour cue, with red indicating Chinese and blue indicating English.

### 3.3. Procedure

Participants were tested individually in a quiet room, and they were seated approximately 40 cm from the laptop screen. Prior to the experiment, participants were required to sign the Participant Consent Form of University of Cambridge. Verbal instructions were then given to them before the experiment that they were supposed to name the digits and the pictures as quickly and accurately as possible in either their L1 or L2 according to the colour cues. Depending on the condition, participants were informed which type of stimuli would be presented on the laptop screen.

Before the formal experiment, participants were required to name each picture stimulus both in Chinese and English without time pressure and given the correct name in the case of an error. In addition, to familiarize the participants with the experiment and the voice-key, they proceeded with a practice block containing 16 trials (8 picture trials and 8 digit trials).

During the experiment, written instructions were presented on the screen in the participants’ native language, Chinese. Then, each trial started with a fixation across (“+”) presented for 400 ms. Then a red or blue square appeared on the screen for 600 ms as a language cue, immediately after which an Arabic numeral or a picture was presented. The stimuli remained on the screen for 1,300 ms during which participants’ reaction times (RTs) were recorded by the SuperLab 6.0. Then, the next fixation across was presented for 400 ms before the subsequent trial began. Participant were given a four-minute break between blocks. The whole experiment took approximately 45 min to compete.

### 3.4. Apparatus

The whole experiment was conducted using a laptop running MicroSoft Windows 10 operating system. Stimulus presentation and data collection were set out using SuperLab 6.0 software (Cedrus Corp.). Naming responses were collected using an Input Microsoft Sound Mapper. Participants’ naming latencies were recorded by the Realtek HD Microphone, which measured from the display of the target stimulus to the speech onset of the vocal responses. The writer sat next to participants to record naming accuracies.

### 3.5. Data coding and analysis

The first trial in each condition was coded as a null switch trial and thus excluded from subsequent analyses. In addition, naming responses beyond the response interval (1,300 ms) or less than 600 ms during which the microphone was mis-triggered (e.g., by stuttering or cough) were excluded from the data analyzes (3.9% of the data). Naming errors here refer to those incorrect names and the inappropriate response language.

The dependent variables were participants’ accuracy rates and naming latencies (RTs). The within-subject independent variables in the basic contrast were the ‘stimulus type’ (digits, the standard pictures), the ‘response language’ (Chinese vs. English), and the ‘language transition type’ (switch vs. stay trials). The mean correct response latencies (RT) and percentage error data were analyzed separately using analysis of variance (ANOVA) run in IBM SPSS Statistics (SPSS Inc. Released 2007. SPSS for Windows, Version 16.0. Chicago, SPSS Inc). In further contrast, participants’ performance in the standard picture set was compared to that in two picture stimulus control conditions: (1) semantic control condition, and (2) repeated number control condition.

It should be noted that switching costs are mainly calculated by the reaction time difference but not the accuracy (or error) rate between stay and switch trials in previous language and task switching studies. This is because accuracy rates were either very high (e.g., Guo et al., 2011) or insensitive to switching costs (e.g., Meuter and Allport, 1999; Costa and Santesteban, 2004). Therefore, this study will follow this trend: the analysis of the accuracy rate data were reported but switching costs were only measured by the naming latency difference between stay and switch trials.

## 4. Data analysis

### 4.1. Digit vs. standard picture stimuli

**Figure 1:**
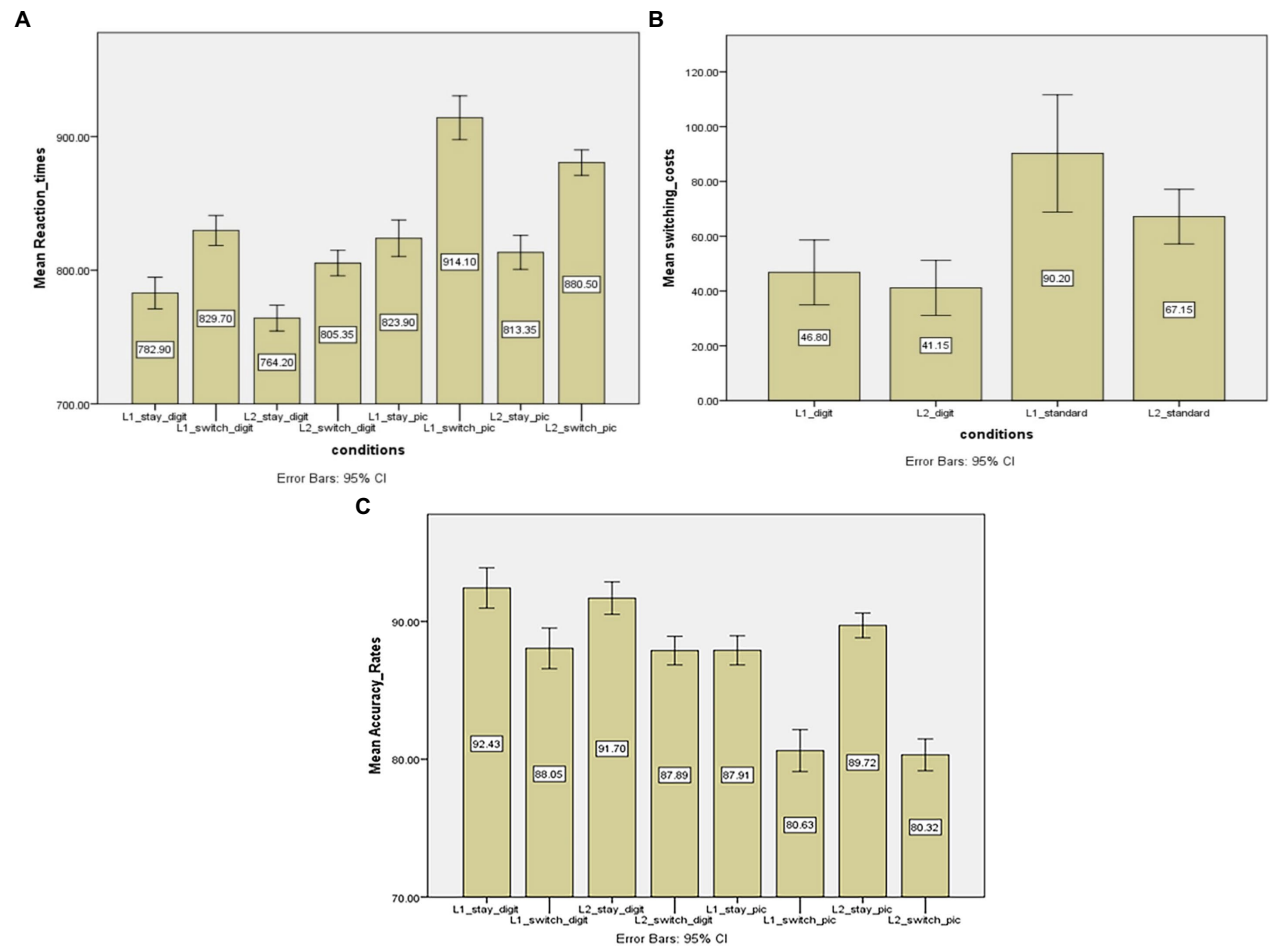
**(A)** Mean reaction times (in ms) of stay and switch trials across two stimulus conditions (digits vs. standard pictures). **(B)** Switching costs (in ms) as a function of ‘response language’ (Chinese vs. English) and ‘stimulus type’ (digits vs. standard pictures). **(C)** Mean accuracy rates (in percentage) of stay and switch trials across two stimulus conditions (digits vs. standard pictures). L1 = the first language, L2 = the second language. Digit = the digit block, pic = the standard picture block.

In this comparison, performance between digits naming and standard picture naming was compared (see Table 1 for a summary of this comparison). Figure 1A shows mean reaction times in different trials. It is important that the two-way interaction effect was found between ‘transition type’ and ‘stimulus type’, *F* (1,19) = 49.506, *p* < 0.001; MSE = 333; *η_p_^2^* = 0.177, revealing that the naming latency differences between switch trials and stay trials varied from Arabic digits to objects. Specifically, the switching costs in digit naming were smaller than those in picture naming (90 vs. 44 ms in picture naming and digit naming, respectively). In contrast, the two-way interaction effects of “response language’*‘stimulus type’, *F* (1,19) = 0.042, *p* > 0.05, and ‘response language’*‘transition type’, *F* (1,19) = 4.083, *p* > 0.05, and the three-way interaction effect of ‘response language’*‘stimulus type’*‘transition type’, *F* (1,19) = 2.646, *p* > 0.05, were not significant.

**Table 1:** Reaction times in ms and accuracy rates in percentage (standard deviations in brackets) in the digit naming and standard picture naming.

	L1 (Chinese)		L2 (English)	
	Stay trial	Switch trial	Stay trial	Switch trial
Digits	782.90 ms (25) 92.43% (3.0)	829.70 ms (24) 88.05% (3.0)	764.20 ms (21) 91.70% (2.1)	805.35 ms (20) 87.89% (2.0)
Standard pictures	823.90 ms (29) 87.91% (2.0)	914.10 ms (25) 80.63% (3.0)	813.35 ms (27) 89.72% (2.0)	880.50 ms (20) 80.32% (2.0)

In addition, further paired sample *t*-tests were performed to examine the switching costs were asymmetrical between two languages in different stimulus type conditions (see Figure 1B for the overall switching costs in different conditions). For the digit naming, there was no significant difference between switching costs of L1 and L2 (46 ms vs. 41 ms in L1 and L2, respectively), *t* (19) = 0.746, *p* > 0.05. For the picture naming, however, L1 resulted in larger switching costs than L2 did (90 vs. 67 ms in L1 and L2, respectively, *t* (19) = 2.247, *p* < 0.05).

In terms of the main effect, there was a significant effect of ‘stimulus type’, *F* (1,19) = 187.471; *p* < 0.05; MSE = 1,752; *η_p_*^2^ = 0.908, suggesting that picture naming was much slower than digits naming (i.e., 764 vs. 854 ms in digit naming and picture naming, respectively). Second, there was a significant effect of ‘response language’, *F* (1,19) = 47.63; *p* < 0.05; MSE = 704; *η_p_^2^* = 0.715, showing that L1 resulted in slower naming latencies than the L2 did (823 vs. 794 ms, respectively). Third, ‘transition type’ also showed a significant effect, *F* (1,19) = 198. 642, *p* < 0.05; MSE = 899; *η_p_^2^* = 0.913, suggesting that naming in switch trials was slower than in stay trials (i.e., 776 vs. 843 ms in stay and switch trials, respectively). This also indicates that robust switching costs were observed in the picture and digit naming tasks when the cue-to-stimulus intervals were at 600 ms.

Figure 1C shows mean accuracy rates in different trials. A two-way interaction effect was observed between ‘stimulus type’ and ‘transition type’, *F* (1,19) = 179.776, *p* < 0.05, MSE = 5.495; *η_p_^2^* = 0.633, suggesting that accuracy rate differences between two types of trials varied across digits and pictures. This result is consistent with the observation of RT analysis that switching costs were different between the picture naming and digit naming. However, main effects of other two-way interaction of ‘response language’*‘transition type’, *F* (1,19) = 0.602, *p* > 0.05, and ‘response language’*‘stimulus type’, *F* (1,19) = 1.904, *p* > 0.05, and three-way interaction, *F* (1,19) = 3.046, *p* > 0.05 were not significant. Additionally, there were significant effects of ‘stimulus type’, *F* (1,19) = 168.057, *p* < 0.05, MSE = 1154.550; *η_p_^2^* = 0.898, and ‘transition type’, *F* (1, 19) = 204.755, *p* < 0.05, MSE = 1545.049; *η_p_^2^* = 0.915. This shows that switch trials resulted in more errors than stay trials did, which is in line with the RT analysis that switch trials caused slower naming responses. On the other hand, the variable ‘response language’ did not show the significant effect, *F* (1,19) = 0.9, *p* > 0.05, suggesting that both L1 and L2 caused comparable naming errors.

### 4.2. Discussion

The purpose of this comparison (digits vs. standard pictures) is to examine whether the stimulus type could have a potential influence on switching costs. Replicating previous studies on language switching (e.g., Meuter and Allport, 1999; Costa and Santesteban, 2004; Costa et al., 2006; Philipp et al., 2007), switch trials resulted in slower naming latencies and lower accuracy rates in digit and picture naming, that is, the switching costs were clearly observed. This result is consistent with the IC model (Green, 1998) that the presence of switching costs is due to the effort to overcome the inhibition of the previously non-target language. In addition, crucial to the comparison between two different types of stimuli is that the amounts of switching costs are modulated by the stimulus type as shown in the interaction effect between the ‘transition type’ and ‘stimulus type’. In other words, it was found that the switching costs were smaller in the digit naming than in the picture naming, which is in line with the Hypothesis 1 that the picture naming will lead to larger switch costs compared to the digit naming. Surprisingly, the stimulus types also had an effect on the asymmetry of switch cost; symmetrical switching costs were observed in digit naming not in standard picture naming.

The observed digit effect is a novel finding showing that the stimulus type can have a potential effect on the size and (a)symmetry of switching costs, given that the previous literature on language switching has largely focused on examining participant-related factors, such as language proficiency or the age of the L2 acquisition, that might affect the switching costs (e.g., Costa and Santesteban, 2004; De Groot and Christoffels, 2006; Festman and Mosca, 2016). This observed “digit facilitation effect” could explain the disparities in switch costs observed in previous studies using these two types of stimuli.

As aforementioned, the Arabic digits have two unique characteristics (the phonological overlap is not applicable in Chinese-English language combination): (1) they belong to the same semantic category group; (2) they are repeatedly presented, compared to the standard objects, which might be attributed to the smaller size and symmetry of switching cost differences. This argument appears to be rational considering Costa and Santesteban (2004) and Costa et al. (2006), where symmetrical switching costs were also reported with only ten pictures being repeatedly named through the whole experiment. Therefore, in order to examine whether the reduction in switching costs is due to these characteristics, participants’ performances in the semantic picture set and repeated picture set were analyzed in the following.

### 4.3. The standard picture set vs. the semantic control set

In this comparison, participants’ performance in the standard picture set was compared to that in the semantic control stimuli set. Similar to the previous comparison, a three-way 2 (response language: Chinese vs. English)*2 (language transition type: stay vs. switch trials)*2 (stimulus type: the standard vs. semantically related pictures) repeated ANOVA was performed for RT and accuracy rates analyses. Table 2 highlights participants naming performance in different conditions.

**Table 2:** Reaction times in ms and accuracy rates in percentage (standard deviations in brackets) in the semantically related picture naming and standard picture naming.

	L1 (Chinese)		L2 (English)	
	Stay trial	Switch trial	Stay trial	Switch trial
Semantically-related pictures	846.55 ms (32) 85.34% (2.0)	884.75 ms (35) 85.08% (2.0)	832.85 ms (29) 84.06% (3.0)	852.60 ms (34) 83.98% (2.0)
Standard pictures	823.90 ms (29) 87.91% (2.0)	914.10 ms (25) 80.63% (3.0)	813.35 ms (27) 89.72% (2.0)	880.50 ms (20) 80.32% (2.0)

A two-way interaction effect between ‘language transition type’ and ‘stimulus type’ was observed, *F* (1, 19) = 36.147; *p* < 0.05; MSE = 683.347; *η_p_^2^* = 0.655, suggesting that naming latency differences between switch trials and stay trials were different between two stimulus types. Specifically, the switching costs were larger in the standard picture set than in the semantic control set (79 vs. 30 ms, respectively). Therefore, it can be argued that semantic information of the stimulus can account for the decrease in the switching costs. Additionally, the interaction effect of ‘response language’ and ‘transition type’ (*F* (1,19) = 5.904, *p* < 0.05, MSE = 845.309, *η_p_^2^* = 0.211) indicates that switching costs were different between the L1 and the L2. In contrast, the two-way interaction effects of ‘response language’*‘stimulus type’, *F* (1,19) = 0.009, *p* > 0.05, and the three-way interaction effect, *F* (1,19) = 0.056, *p* > 0.05 were not significant (see Table 2 for a summary of this comparison).

**Figure 2:**
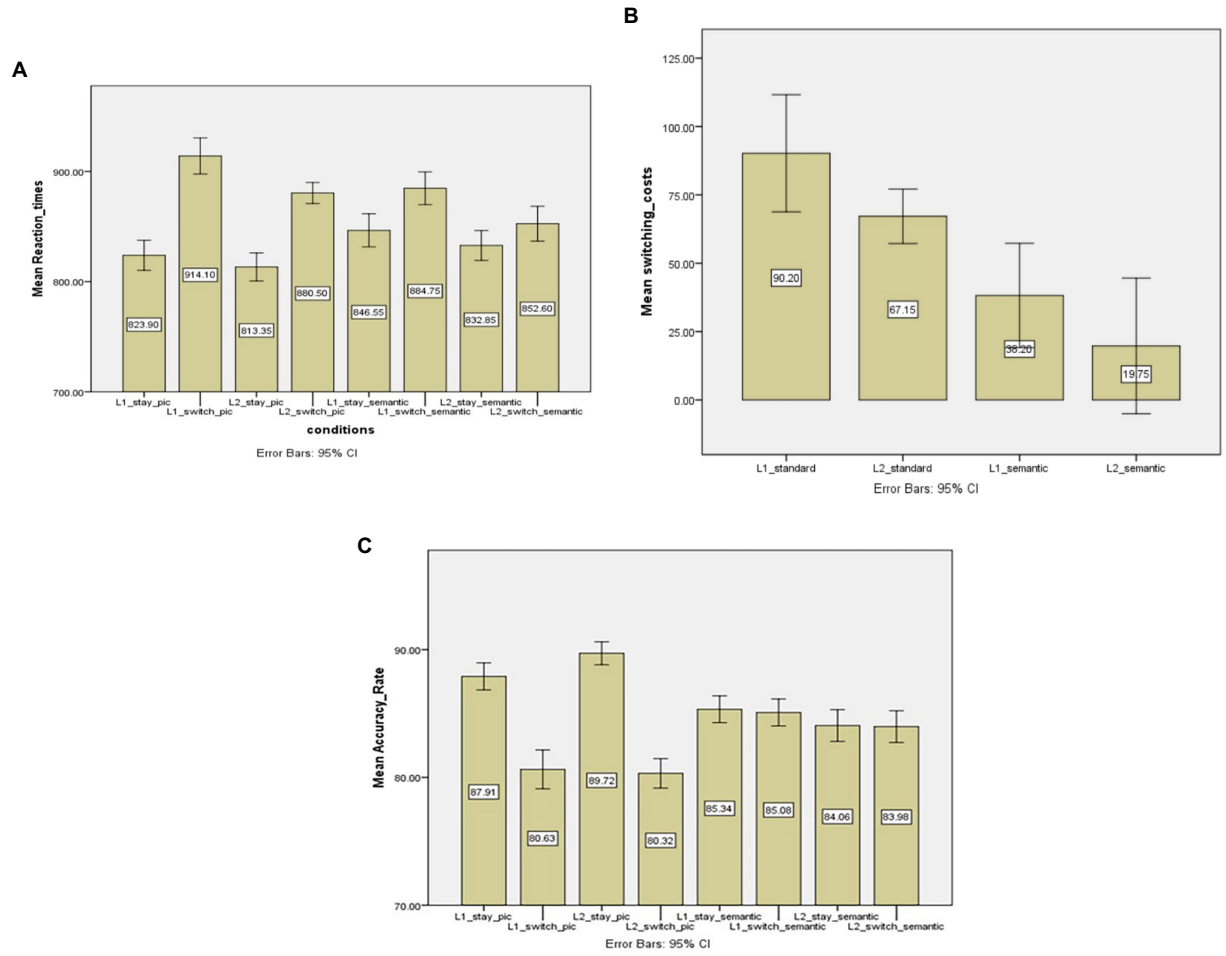
**(A)** Mean reaction times (in ms) of stay and switch trials across two stimulus conditions (standard vs. semantically related pictures). **(B)** switching costs (in ms) as a function of ‘response language’ (Chinese vs. English) and ‘stimulus type’ (standard vs. semantically related pictures). **(C)** Mean accuracy rates (in percentage) of stay and switch trials across two stimulus conditions (standard vs. semantically related pictures). L1 = the first language, L2 = the second language. Standard = the standard picture condition, semantic = the semantically related picture condition.

In order to further examine the effect of stimulus type on the asymmetry in switching costs, paired sample t-tests were performed (see Figure 2B for the overall switching costs in different conditions). For the standard picture naming, the switching costs for the L1 were significantly larger than those for the L2 (90 vs. 67 ms in L1 and L2 naming, respectively), *t* (19) = 2.247, *p* < 0.05. However, in the semantic control set, the switching costs were comparable for L1 and L2 (38 vs. 20 ms), t (19) = 1.160, *p* > 0.05. Therefore, this pattern of results assumes that the semantic blocking effect can modulate the asymmetry in switching costs.

According to Figure 2A, naming responses in switch trials were significantly slower than those in stay trials (829 vs. 882 ms; *F* (1, 19) = 112.050; *p* < 0.05; MSE = 1034.255; *η_p_^2^* = 0.855), pointing to the switching costs. Additionally, there was also a main effect of ‘response language’, *F* (1, 19) = 67.601; *p* < 0.05; MSE = 299.553; *η_p_^2^* = 0.781, suggesting that the L1 resulted in slower naming latencies than the L2 did (867 vs. 844 ms in L1 and L2 naming, respectively).

Figure 2C shows mean accuracy rates in different trials. There was a two-way interaction effect of the ‘transition type’*‘stimulus type’, *F* (1,19) = 76.609, *p* < 0.05, MSE = 666.672; *η_p_^2^* = 0.801, suggesting that the accuracy rate differences between stay and switch trials were larger in the standard picture naming than in the semantically related picture naming. This is in line with the observation of the naming latencies, confirming that the switching costs would become smaller in the semantic blocking condition. Other two-way interaction effects of ‘response language’ * ‘stimulus type’ (*F* (1,19) = 3.933, *p* > 0.05) and ‘response language’ * ‘transition type’ (*F* (1,19) = 1.567, *p* > 0.05) were not significant. Lastly, there was no three-way interaction effect, *F* (1,19) = 2.681, *p* > 0.05. The analysis of accuracy rates also showed that the main effect of the ‘transition type’, *F* (1,19) = 115.309, *p* < 0.05, MSE = 723.350; *η_p_^2^* = 0.859, was significant, suggesting that switch trials caused more errors than stay trials did. In contrast, main effects of the ‘response language’, *F* (1,19) = 0.297, *p* > 0.05, and the ‘stimulus type’, *F* (1,19) = 0.052, *p* > 0.05, were not significant.

### 4.4. Discussion

The results clearly reflect the influence of stimulus type on language switching costs, that is, the switching costs can be modulated when stimuli belong to the same semantic category, which provides evidence for the Hypothesis 2. Specifically, the switching costs became smaller in the semantic control set in which the semantic blocking condition was formed than in the standard picture set. In addition, the asymmetry in switching costs was also affected, that is, 1 caused the same amount of switching costs as the L2 did in the semantic control picture set.

Crucial to the present context is the observation that the speed with which a given picture can be named is affected by whether or not objects from the same category have been named in the preceding trials (e.g., Damian et al., 2001; Rahman and Melinger, 2009). Previous studies on blocked naming task manipulate the context in which pictures appear in successive trials depicting objects that are from the related semantic categories—the homogeneous context, or that are not related to each other—the heterogeneous context. The general finding is that objects are named more slowly in the homogenous context than in the heterogeneous context, when pictures are named several times (e.g., Damian et al., 2001; Damian and Rahman, 2003) or once (e.g., Kroll and Stewart, 1994). This finding may predict that stay trials in the semantic control set would result in slower naming responses than those in the standard picture set due to the semantic blocking effect. To such an end, a 2 (response language: Chinese vs. English) * 2 (stimulus type: the standard picture vs. semantically related picture) ANOVA was performed in stay trials. There was a main effect of ‘stimulus type’, *F* (1,19) = 8.936, *p* < 0.05, which confirms the prediction that stay trials in the semantic condition caused slower naming latencies than those in the standard picture condition did (839 vs. 818 ms).

The situation seems to be more complicated if a semantic competitor that has been named previously is in another language. As aforementioned, the IC model (Green, 1998) implements language control in bilingual context through a mechanism that inhibits activation from the language that is not relevant for speech production. In the language staying context, when a language task schema is maintained (e.g., speaking in L1), this inhibitory mechanism suppresses co-activated lemmas from the non-response language (L2). Consequently, this “staying relationship” between two consecutive trials can allow for the within-language semantic interference effect as suggested before, thus causing slower naming latencies in stay trials when objects are semantically related. However, if there is a change of language (e.g., switching from language A to language B in the switch trials) this inhibition mechanism suppresses those lemmas with non-response language A tags that are previously activated for production. Therefore, it can be argued that the inhibition is thought to have a global effect on the non-response language, which means that it will inhibit active words with incorrect language tag (e.g., Green, 1998).

In sum, the semantic blocking effect could cause the interference effect on the stay trial but cannot affect the switch trial, therefore a reduction in switching trials could be reasonably observed when the stimulus belongs to the same semantic category. Furthermore, another interpretation of such a reduction in switching costs and the change in asymmetry might come from the ‘concept-switching facilitation’ theory proposed by recent researcher on language switching studies (e.g., Declerck et al., 2013, 2015; Zhang et al., 2020).

### 4.5. The standard picture set vs. the repeated control set

In the last comparison, participants’ naming responses in the standard picture naming were compared to those in the repeated picture naming. This comparison aims to explore whether the repetition priming effect can lead to the smaller switching costs as predicted in the Hypothesis 3. A 2 (response language: Chinese vs. English) * 2 (language transition type: stay vs. switch trials) * 2 (stimulus type: standard vs. repeated pictures) within-subject ANOVA was performed for RT and accuracy rate analyses. Table 3 highlights participants naming performance in this comparison.

**Table 3:** Reaction times in ms and accuracy rates in percentage (standard deviations in brackets) in the repeated picture naming and standard picture naming.

	L1 (Chinese)		L2 (English)	
	Stay trial	Switch trial	Stay trial	Switch trial
Repeated pictures	783.70 ms (31) 91.65% (2.0)	841.00 ms (39) 90.81% (1.9)	771.85 ms (30) 91.38% (2.0)	798.55 ms (38) 90.86% (2.0)
Standard pictures	823.90 ms (29) 87.91% (2.0)	914.10 ms (35) 80.63% (3.0)	813.35 ms (27) 89.72% (2.0)	880.50 ms (20) 80.33% (2.0)

**Figure 3:**
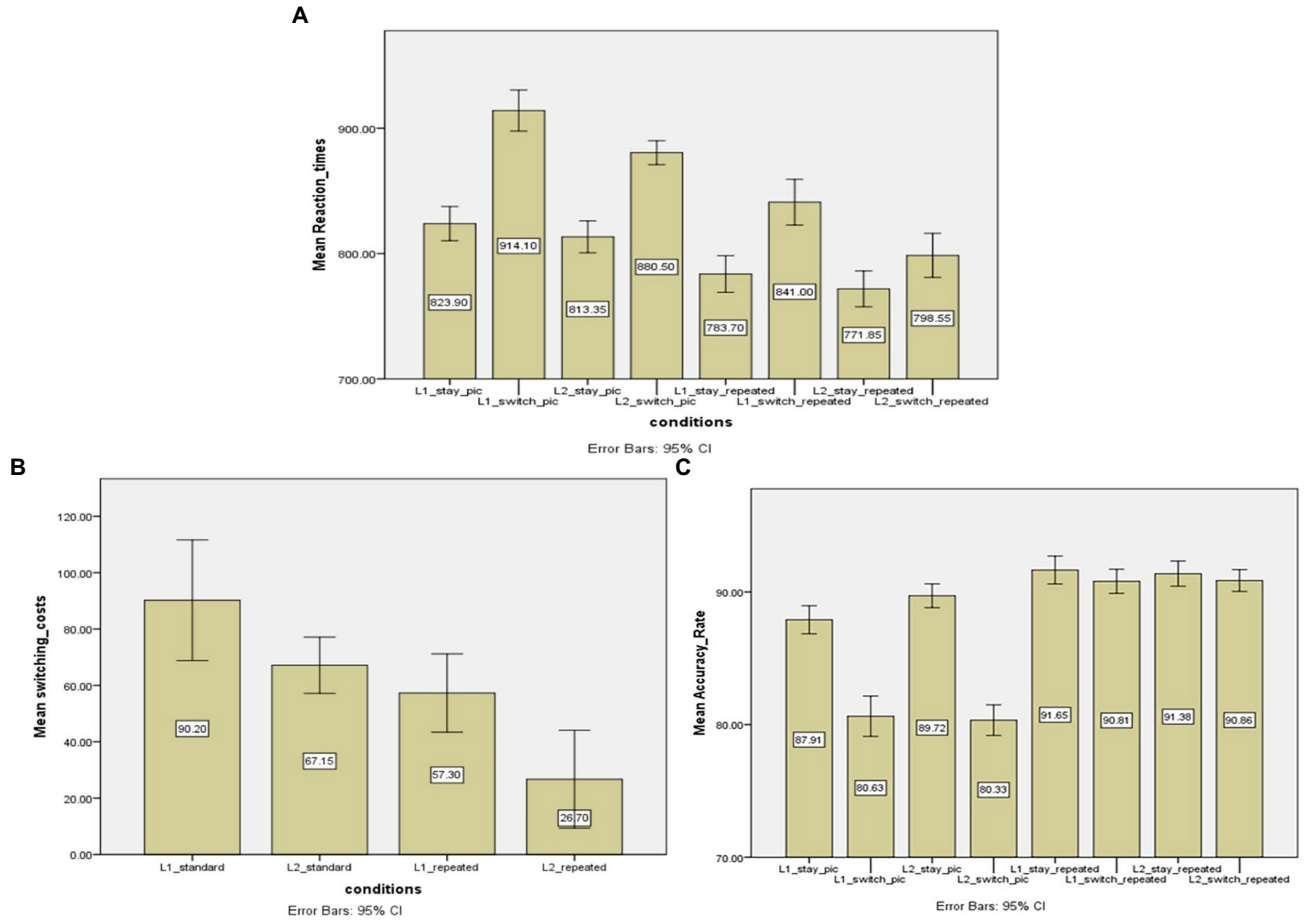
**(A)** Mean reaction times (in ms) of stay and switch trials across two stimulus conditions (standard vs. repeated pictures). **(B)** Switching costs (in ms) as a function of ‘response language’ (Chinese vs. English) and ‘stimulus type’ (standard vs. repeated pictures). **(C)** Mean accuracy rates (in percentage) of stay and switch trials across two stimulus conditions (standard vs. repeated pictures). L1 = the first language, L2 = the second language. Standard = the standard pictures, repeated = repeated pictures.

Figure 3A shows mean reaction times in different trials. A two-way interaction effect was found between ‘transition type’ and ‘stimulus type’, *F* (1,19) = 16.782, *p* < 0.05; MSE = 801.504; *η_p_^2^* = 0.469, revealing that the naming latency differences between switch trials and stay trials varied from the standard picture set to the repeated control set. Specifically, the switching costs in the repeated picture naming were smaller than those in the standard picture naming (42 vs. 79 ms). In contrast, the two-way interaction effect of “response language’ * ‘stimulus type’ (*F* (1,19) = 0.250, *p* > 0.05) was not significant. Lastly, there was a three-way interaction effect of ‘response language’, ‘stimulus type’ and ‘transition type’, *F* (1,19) = 5,933, *p* < 0.05, MSE = 379.250; *η_p_^2^* = 0.238, suggesting that switching cost differences between two stimulus types varied from L1 to L2.

In order to further examine whether the stimulus type affects the asymmetry in switching costs, paired sample t-tests were performed (see Figure 3B for the overall switching costs in different conditions). The result showed that the switching costs were asymmetrical between two languages in the repeated picture naming, *t* (19) = 2.779, *p* < 0.05, and the L1 resulted in slower naming responses than the L2 did (57 vs. 27 ms). The same pattern of result was also observed in the standard picture naming, and the switching costs for the L1 were significantly larger than those for the L2 (90 vs. 67 ms), *t* (19) = 2.247, *p* < 0.05. Consequently, it can be argued that the asymmetry in switching costs cannot be modulated by repetition priming effect.

In terms of the main effect, there was a significant effect of ‘stimulus type’, *F* (1,19) = 104.771; *p* < 0.05; MSE = 1337.459; *η_p_^2^* = 0.846, suggesting that the standard picture naming was much slower than the repeated picture naming (857 vs. 798 ms). Second, there was a significant effect of ‘response language’, *F* (1,19) = 16.171; *p* < 0.05; MSE = 1498.454; *η_p_^2^* = 0.460, showing that L1 resulted in slower naming latencies than the L2 did (840 vs. 816 ms, respectively). Third, ‘transition type’ also showed a significant effect, *F* (1,19) = 309.007, *p* < 0.05; MSE = 471.267; *η_p_^2^* = 0.942, suggesting that naming in switch trials was slower than in stay trials (i.e., 798 vs. 858 ms in stay and switch trials, respectively).

Figure 3C shows overall accuracy rates of this comparison. a two-way interaction effect of ‘transition type’ and ‘stimulus type’ was observed here, *F* (1,19) = 107.674, *p* < 0.05, MSE = 584.078; *η_p_^2^* = 0.850, suggesting that accuracy rate differences are significant between two types of stimuli. This is in line with what was observed in the analysis of RT data that switching costs became smaller in the repeated picture naming condition. On the other hand, other two-way interaction effects, such as ‘response language’ * ‘stimulus type’ (*F* (1,19) = 1.320, *p* > 0.05) and ‘response language’ * ‘transition type’ (*F* (1,19) = 1.391, *p* > 0.05) and three-way interaction effect were not significant (*F* (1,19) = 3.207, *p* > 0.05). The results of the accuracy rate analysis also showed that there were main effects of ‘transition type’ (*F* (1,19) = 178.069, *p* < 0.05, MSE = 812.252; *η_p_^2^* = 0.904) and ‘stimulus type’ (*F* (1,19) = 529.405, *p* < 0.05, MSE = 1704.983; *η_p_^2^* = 0.965), but not of ‘response language’ (*F* (1,19) = 4.193, *p* > 0.05). These revealed that stay trials and repeated picture stimuli resulted in higher accuracy rates.

### 4.6. Discussion

In line with the observations of the semantically related vs. standard pictures, the results of RT and accuracy rate analyses here reflect the influence of the repetition priming on language switching costs, that is, the switching costs can be modulated when stimuli are presented repeatedly, which provides evidence for the Hypothesis 3. Specifically, the switching costs became smaller in the repeated control set than in the standard picture set. However, the asymmetry in switching costs appeared not to be affected by the repetition priming effect, since the L1 caused larger switching costs than the L2 did with both types of stimuli. This pattern of results provides clear evidence that the main difference between bilingual picture naming and digit naming in switch paradigm lies to the repetition of digits. The reason why repetition priming effect can modulate language switching costs will be analyzed in the General Discussion section.

## 5. General discussion

### 5.1. Arabic digits vs. pictures: The cognate effect?

It should be noted two languages examined in the present study are Chinese and English that belong to two different language families. Specifically, Chinese belongs to the Sino-Tibetan language family that does not have an alphabet system but uses a logographic system for the written language, and thus Chinese words are not created out of letters as is the case with alphabetic systems (e.g., English and German). This phonological difference will not give rise to the cognate words. Cognates are words in two languages that have a common origin and thus are similar or identical and have the same meaning.

Interestingly, this cognate effective was observed in Declerck et al. (2012) with German-English bilinguals. They hypothesized that many digits (in German and English) are cognate such as “six,” “nine,” “five” in English and “sechs,” “neun” “funf” in German, which can possibly be taken to interpret the difference in switch costs observed in previous studies using different target stimuli. To test their hypothesis, a cognate picture set that constituted of items depicting cognates was added and participant’s naming responses performance in digit naming, standard picture naming and cognate picture naming were recorded. The results showed that switch costs were smaller for digit naming when comparing the digit stimuli set and standard picture stimuli set, while no switch cost difference was observed between the cognate picture set and digit set. Taken together, this data pattern reveals that phonological overlap between two languages can account for the smaller switch costs in digit naming. Furthermore, as suggested by the inhibitory control model, lexical selection mechanism suppresses the activation of the unintended language and the re-activation of previously inhibited lexical items requires time, which accounts for the reason why slower response latencies are observed when switching between two languages. Following this line of logic, if the switch costs become smaller and overcoming the inhibition requires less time, it can be assumed that bilingual digit naming exerts less language control than picture naming. This is arguably because repetition priming effect observed in the present study and phonological overlap reported in Declerck et al.’s (2012) study strengthen the activation levels of target lexical items to reach the threshold for language production, thus partially eliminating the lexical repetition between two languages.

However, the finding that cognate status could reduce the switch costs has been challenged by Verhoef et al. (2009), who examined Dutch-English bilinguals’ performance in the language-switching task and did not observe differences in switch costs between pictures that have cognate words in the other language and those that do not have. Interestingly, Christoffels et al. (2007) even found that pictures depicting cognates exert larger switch costs than those depicting non-cognates. Taken together, whether the cognate can be taken as an indicator of smaller switch costs needs further examination. These contradicted results might reveal that the observation in Declerck et al.’s (2012) study that cognate pictures could reduce the switching costs was mainly due to the repetition of these cognate pictures rather than the phonological overlaps.

### 5.2. Semantic and repetition priming effects and their implications for bilingual language control

As suggested in this experiment, the stimulus type differences could have potential influences on the switching costs, that is, digit naming leads to less magnitude of and symmetry in switching costs than object naming, suggesting that bilingual digit naming requires less inhibitory control than picture naming. Given that this digit-leading facilitation effect is a novel finding (to the best of my knowledge, only one study by Declerck et al. (2012) reached a similar conclusion), there is no such existing theoretical model or framework in the bilingual language production literature that can be taken as an interpretation for it. However, fortunately, the observations through comparing Arabic digits to semantic control picture sets and repeated control picture sets provide a clear hint as to the role of repetition and semantic priming effects in the modulation of the bilingual language control process. Note that this study did not compare digit naming directly with manipulated picture naming is because digits have two different features that makes it difficult to independently test each of them. The second concern is that these two features might cause an additive effect. There are three interpretations for the modulation of semantic priming effect on switching costs.

The first possible reason for a reduction in the switching costs would be a slower naming latency in stay trials if the naming latencies of the switch trials keep constant. This might be the case in the semantic control picture set. Crucial to the semantic contextual effects is the observation that naming latencies with which a stimulus was named was influenced by previous one from the same semantic category. Previous studies on the lexical retrieval process in speech production have had a converging result showing that “retrieving a word has a negative effect on the subsequent retrieval of other words from the same semantic category” (e.g., Oppenheim et al., 2010, p. 227). These negative consequences have been termed cumulative semantic interference. For example, naming ‘dog’ could result in a slower naming latency when it is followed by a semantically related word such as ‘cat’ or ‘pig’ than by an unrelated word such as ‘pen’. Consequently, stay trials in semantic control block may form the cumulative semantic interference effect, leading to slower naming latencies.

However, in switch trials, where the language membership changes, it is argued that the semantic contextual effect should disappear because of the language alternation (e.g., Green, 1998; Runnqvist et al., 2012). As suggested by Green (1998), the bilingual control mechanism achieves inhibition by regulating the so-called “language task-schema” that is responsible for controlling output goals (i.e., speak in the L1 or speak in the L2). These language task schemas control the activation levels of the lexical system by connecting them with language tags that reflect the language membership of the lexicon. In this way, the language task-schema could exert the suppression signal on the lexical system, inhibiting all lexical nodes containing language tags of the non-target language. This inhibitory control process has a globally negative effect on the non-target language tags, suppressing any lexical representation containing incorrect language tags, regardless of any linguistic relationship among non-target lexical items.

Therefore, these two assumptions have led scholars to argue that lexical items that have been previously retrieved in one language will not have sufficient activation to interfere language production when naming semantically-related words in the other language in the following trials (Runnqvist et al., 2012). For example, naming ‘cat’ will not cause interference with the naming of ‘gou‘[dog] in Chinese. As Green (1998, p. 75) explained, “if there is any changes of language, then any lemmas in the previously active language will become inhibited.” In certain circumstances, this should lead to the abolition of both across-language and within-language competition priming.” This argument was confirmed in Lee and Williams’s (2001) study, where they found that the semantic interference effect disappeared when there was a change of language membership in advance of the production of the target stimulus. Taken together, this line of evidence suggests that the semantic status of the picture stimuli does not have any effect on the language alternation or switch process.

In sum, the reduction in switching costs could be attributed to the different functionality of semantic contextual interference in stay and switch trials. On the other hand, the RT data seem to be at odds with a strong version of the IC model that predicts that the semantic contextual interference effect could be completely abolished since there were still slower naming latencies in switch trials in the semantic control block compared to those in the standard picture block. Nevertheless, the weaker version of the IC model still allows for some components of the semantic interference effects when switching between languages might fit with the present study.

The second possible explanation of the finding that semantic priming effects allow for reducing the need for inhibitory control comes from the argument that bilingual language control might occur at different lexical processing stages (e.g., Green, 1998; Declerck et al., 2015; Zhang et al., 2020). As explained in the literature review section, the second tenet of the IC model argues that inhibitory control is reactive, suggesting that the stronger the activation of competitors in the non-target language, the stronger the inhibition that needs to be applied. Following this logic, an observed reduction in the inhibition process could imply that the activation of lexical representations in the unintended language is affected by some other factors, such as the semantic and phonological relationships between words.

According to the language production model (e.g., Levelt et al., 1999), language production starts with the activation of semantic representations that then spreads to lexical nodes at the lemma level, followed by activation at the phonological level. For example, when bilinguals switch from “dog” in English to the “cat” in Chinese, it requires switching between semantic representations (or concepts, i.e., from “DOG” to “CAT”) and lexical representations (or lemmas, i.e., from “dog” to “猫” cat in Chinese). One could argue that manipulations at the concept level should affect language switching (e.g., Declerck et al., 2013, 2015; Chang et al., 2016). This appears to be the case in the semantic control picture set. Note that semantically related pictures were presented in a blocked condition (i.e., animal, occupations, and transportation blocks), allowing participants to be aware of the semantic categories at the start of each block. Consequently, those semantic representations or concepts (at least some of them) belonging to a specific semantic category group can be prepared or activated in the bilingual’s lexicon, compared to the standard picture condition, where there is no such prepared activation. Consequently, once the semantic representations in the same category can be projected in advance, the switching costs at the concept level are reduced. This argument is congruent with Zhang et al.’s (2020) study, where they found that related and repeated concepts facilitate language switching as compared to unrelated concepts.

This prepared activation caused by the conceptual facilitation effect reduces the amount of inhibition needed to suppress the non-target semantic representations, thus speeding up the concept selection process. This argument appears to be reconciled with the IC model in a way that inhibitory control is still required globally to suppress the activation of non-target representations and lemmas. Furthermore, it might also be that participants may notice the shared semantic feature of the items in the blocked condition as in this experiment and use this knowledge to predict other items in this semantic cohort (Belke et al., 2017). That is, the semantic priming effect constrains the activation of lexical items to a certain semantic category, which may lead to a local inhibition of non-target lexical items belonging to this semantic group. Compared to the global inhibition executed on the entire language, this local inhibition might be much weaker so that switching costs become smaller when pictures are semantically related. Taken together, these could suggest that different conditions might invoke different types of inhibitory control, i.e., local versus global control.

According to Declerck et al. (2015) and Kroll and Stewart’s (1994) Concept-Word Association Model, once the semantic concepts are activated, L1 lexical nodes receive a higher level of activation than those of L2 because the concepts have a stronger connection to their L1 lemma than their L2 translation equivalents. Therefore, in the semantic control block, where the conceptual representations can be predicted and prepared, L1 lexical representations will receive more activation than L2 lexical items. Furthermore, as Declerck et al. (2015) argued, this effect only operates on switch trials, where cross-language competition is fierce. Therefore, when switching from L2 to L1, this extra-activation of L1 lexical representations in the semantic control block makes the recovery from inhibition of L1 lemmas much easier and less time-consuming than the standard picture block. In contrast, when switching from L1 to L2, the L2 lemmas are activated less than the L1 lemmas, prior to the language control process, and thus should be more difficult to recover. These activation differences therefore reduce the asymmetry in switching costs in the semantic control set compared to the standard picture set. Furthermore, this idea seems to illustrate that language control mechanisms may involve both inhibition and facilitation (Declerck et al., 2013).

Regarding the repetition priming effect, Kleinman and Gollan (2018) observed that naming pictures in one language slowed the subsequent naming of their translation equivalents, such that naming ‘dog’ inhibits ‘perro’ and vice versa to the same extent. Moreover, they also found that this inhibitory effect is considered long-term, and “not only does this inhibition effect over trials, which also accumulates without plateauing for at least as long as measured here in; i.e., 96 trials’ (Kleinman and Gollan, 2018, p. 122). These patterns of results thus led them to further assume that the cross-language repetition priming effect in fact results in an increase in switching costs but does not affect the asymmetry of switching costs.

At first glance, this argument in a sense is in direct conflict with repetition priming and the results observed from the repeated picture control group. In addition, the idea that this long-term inhibitory effect resulted from a cross-language repetition effect also contradicts what was found in Francis and Saenz, 2007 study. It was found that Spanish–English participants named picture stimuli more quickly in both L1 and L2 when they had previously named the same pictures than when they have never named them. Here, it is very difficult to deny Kleinman and Gollan’s (2018) argument for lateral inhibition of the translation equivalent, thus I adopt a compromise to interpret it. Specifically, the co-existence of between-language inhibition and repetition facilitation raises the possibility that the repetition facilitation effect from previously naming the stimuli can dwarf the inhibition of naming it in the other language.

In sum, in the present study it is clear that the inhibitory control mechanisms can be modulated by other aspects of language processing such as repetition and semantic priming effects. However, there is no model of inhibitory control specified in previous studies that can interpret these patterns of findings, and thus future research needs to work on this field to examine how bilingual language control is adapted to different language processing conditions. Furthermore, note that this study adopted a cued (forced) language switching paradigm where the response language is determined by the colour cue, which is significantly different from voluntary language switching (e.g., natural naming). Some studies using a voluntary language switching task (e.g., Gollan and Ferreira, 2009; Blanco-Elorrieta and Pylkkanen, 2017; de Bruin et al., 2018) reported that language switching costs became smaller or even disappeared in the voluntary language switching task compared to forced (cued) language switching. This, in combination with their neuroimaging evidence, leads Blanco-Elorrieta and Pylkkanen (2017) to suggest that inhibition may not be required when bilinguals spontaneously switch between two languages and that a language-specific selection mechanism allowing bilinguals to directly select target lexical items regardless of robust lexical competition is developed. Nevertheless, considering the current findings that inhibitory control may operate at different levels [e.g., semantic representation level (conceptual level), lemma (lexical selection) level, phonological level, and so on], it might be that voluntary language switching allows for the workaround of some components of inhibition (but not all of them) associated with language switching at certain levels. This argument appears to be consistent with findings that switching costs became smaller but did not disappear as observed by Gollan and Ferreira (2009). Furthermore, it is still unclear how and where voluntary language switching reduces switching costs and modulates inhibitory control processes, which deserves further investigation.

## 6. Conclusion

Previous language switching studies mainly used two types of stimuli: Arabic digits and pictures, however, the results appear to be inconsistent: the size of and (a)symmetry in switching costs differ across studies. The current experiment was designed to examine whether and how these methodological differences (task-level factors) modulate the size of and (a)symmetry in switching costs. The results revealed that the digit naming resulted in smaller switching costs than the picture naming, suggesting that bilingual language control is less required in digit naming. However, language switching costs became smaller, when picture stimuli belonged to the same semantic categories or were repeated presented throughout the experiment, compared to when they were unrelated and unrepeated. These results further suggested that semantic and repetition priming effects helped to reduce switching costs and explain inconsistent results of switch costs in previous studies using different types of stimuli. It is further argued that semantic priming effects can result in less global inhibition required to suppress the activation of non-target language and a local inhibitory control executed only on a specific semantic category.

In general, these results are in line with Green’s (1998) inhibitory control model that bilingual language production requires the inhibition of the non-target language, and the extent of this inhibition depends on the proficiency (or dominance) of the non-target language, that is, the dominant L1 is inhibited to a larger extent than the weaker L2.

### 6.1. Limitation

Considering a random selection of participants in this study and previous language switching literature, a power analysis should be conducted in future work as a more scientific and rigorous way to determine the sample size. The second deficiency is that participants’ L2 proficiency was mainly indicated by interviews and their IELTs scores, and these qualitative measurements are insufficient. Hence, standardized measurements such as the LEAP-questionnaire (Kaushanskaya et al., 2020) and the Multilingual Naming Test (Gollan et al., 2012) can be used in future research to examine participants’ L2 proficiency and socio-cultural status quantitatively.

## Data availability statement

The original contributions presented in the study are included in the article/Supplementary material, further inquiries can be directed to the corresponding author.

## Ethics statement

The studies involving human participants were reviewed and approved by the Department of Theoretical and Applied Linguistics Ethics Committee (DTAL) at the University of Cambridge. The participants provided their written informed consent to participate in this study.

## Author contributions

QS designed and conducted the study, completed the statistical analysis, and wrote the manuscript. YC managed the figure information, co-wrote the manuscript, and contributed to manuscript revision. Both authors contributed to the article and approved the submitted version.

## Conflict of interest

The author declares that the research was conducted in the absence of any commercial or financial relationships that can be construed as a potential conflict of interest.

## Publisher’s note

All claims expressed in this article are solely those of the authors and do not necessarily represent those of their affiliated organizations, or those of the publisher, the editors and the reviewers. Any product that may be evaluated in this article, or claim that may be made by its manufacturer, is not guaranteed or endorsed by the publisher.

## Footnotes

^1^All picture stimuli used in the current study obtained from: https://crl.ucsd.edu/experiments/ipnp/ (Accessed on November 11, 2021).
